# Drug Burden Index Is a Modifiable Predictor of 30-Day Hospitalization in Community-Dwelling Older Adults With Complex Care Needs: Machine Learning Analysis of InterRAI Data

**DOI:** 10.1093/gerona/glae130

**Published:** 2024-05-11

**Authors:** Robert T Olender, Sandipan Roy, Hamish A Jamieson, Sarah N Hilmer, Prasad S Nishtala

**Affiliations:** Department of Life Sciences, University of Bath, Bath, UK; Department of Mathematical Sciences, University of Bath, Bath, UK; Department of Medicine, University of Otago, Christchurch, New Zealand; Faculty of Medicine and Health, Kolling Institute, Northern Clinical School, The University of Sydney and Northern Sydney Local Health District, St Leonards, New South Wales, Australia; Department of Life Sciences & Centre for Therapeutic Innovation, University of Bath, Bath, UK; (Medical Sciences Section)

**Keywords:** Artificial intelligence, Decision tree, Hospitalization, Logistic regression, Predictive modelling

## Abstract

**Background:**

Older adults (≥65 years) account for a disproportionately high proportion of hospitalization and in-hospital mortality, some of which may be avoidable. Although machine learning (ML) models have already been built and validated for predicting hospitalization and mortality, there remains a significant need to optimize ML models further. Accurately predicting hospitalization may tremendously affect the clinical care of older adults as preventative measures can be implemented to improve clinical outcomes for the patient.

**Methods:**

In this retrospective cohort study, a data set of 14 198 community-dwelling older adults (≥65 years) with complex care needs from the International Resident Assessment Instrument-Home Care database was used to develop and optimize 3 ML models to predict 30-day hospitalization. The models developed and optimized were Random Forest (RF), XGBoost (XGB), and Logistic Regression (LR). Variable importance plots were generated for all 3 models to identify key predictors of 30-day hospitalization.

**Results:**

The area under the receiver-operating characteristics curve for the RF, XGB, and LR models were 0.97, 0.90, and 0.72, respectively. Variable importance plots identified the Drug Burden Index and alcohol consumption as important, immediately potentially modifiable variables in predicting 30-day hospitalization.

**Conclusions:**

Identifying immediately potentially modifiable risk factors such as the Drug Burden Index and alcohol consumption is of high clinical relevance. If clinicians can influence these variables, they could proactively lower the risk of 30-day hospitalization. ML holds promise to improve the clinical care of older adults. It is crucial that these models undergo extensive validation through large-scale clinical studies before being utilized in the clinical setting.

Older adults aged ≥65 years are a growing population, which accounts for the highest proportion of hospitalizations and in-hospital deaths (1) and exhibits the highest multimorbidity (2). These factors pressure healthcare systems, older adults requiring additional care, and medical resources. For older adults, hospitalization should be avoided unless necessary (3). Hospitalization increases the chances of patient complications, such as hospital-acquired pneumonia (4), hospital-acquired incontinence (5), deep vein thrombosis (6), pulmonary embolism (7), pressure ulcers (8), falls and related injuries (9), and delirium (10). Identifying patients at high risk of hospitalization can provide opportunities to intervene to prevent unnecessary admissions and to facilitate and resource care for individuals who need treatment in the hospital.

Traditional statistical approaches are parsimonious, have several limiting a priori assumptions, and do not give insight into the importance of each variable for predicting the outcome. Machine learning (ML) models do not suffer these drawbacks (11). There is an immediate need to develop accurate predictive models to identify high-risk individuals and factors associated with an increased risk of unfavorable clinical events, such as mortality and hospitalization (12). ML approaches have been shown to produce highly accurate models predicting clinical outcomes such as mortality (13) and delirium (14) from older adults’ patient data. It should be noted that ML models are not expected to replace traditional statistical approaches or clinical acumen but rather become an additional tool in health service planning and the armamentarium of a skilled clinician.

The true power of ML models lies in identifying modifiable factors highly associated with unfavorable clinical events such as hospitalization. A nonmodifiable risk factor, such as sex or age, can provide insight into the likelihood of an unfavorable clinical event. However, although helpful for planning health services or prioritizing patients for clinical review, this information is of little relevance to individual clinical management. On the contrary, it has been shown in multiple studies that identifying modifiable risk factors provides great clinical insight into clinicians, ultimately leading to a lowered risk of unfavorable clinical events (15–17). Inappropriate medication use, defined as the use of medications where the current harm outweighs the benefit for the individual, is a modifiable risk factor closely associated with increased hospitalization rates and mortality in older adults (18,19). The Drug Burden Index (DBI), a validated metric, quantifies an individual’s exposure to anticholinergic and sedative medications (20). The DBI is a practical measure of cumulative risk of medication-related functional impairment in older adults (21), which is an important clinical consideration when identifying inappropriate polypharmacy. Given that modifiable risk factors such as the DBI can be identified, this can directly inform clinical care of older adults (22), improving clinical outcomes and minimizing the risk of unfavorable clinical events.

This study assesses the importance of several risk factors in predicting 30-day hospitalization in older adults aged 65 years and over with complex care needs. Identifying modifiable risk factors associated with hospitalization could tremendously affect geriatric care and future direction regarding models of care to reduce unnecessary hospitalizations and prescribing guidelines in older adults (23).

This study aimed to:

1. Develop 3 ML classification models to predict 30-day hospitalization.
2. Evaluate 3 ML classification models in terms of discriminatory power.
3. Identify important modifiable risk factors in predicting 30-day hospitalization.

Three ML classification models were developed as part of this study: Random Forest (RF), XGBoost (XGB), and Logistic Regression (LR). RF is a widely used ML approach that combines the output of multiple decision trees to reach a single result (24). XGB is a gradient-boosting algorithm. Although RF trains multiple trees in parallel and outputs the most common outcome, XGB creates a sequential ensemble of improved tree models to predict the outcome (25). LR describes the relationship between a dependent binary outcome variable and several independent input variables (26).

## Method

The Academic Ethics and Integrity Committee at the University of Bath has approved this project (Form No: 6738). The data set utilized in this study was the InterRAI Home Care Assessment System repeat assessment at baseline. InterRAI is a collection of instruments utilized internationally to provide clinicians with insights into a patient’s individual care requirements. In New Zealand, the Home Care assessment (InterRAI-HC) is employed to assess individuals with complex care needs looking to enter publicly funded aged residential care, whereas the shorter Contact assessment is utilized for those seeking home-based support services. Both assessments encompass a range of questions that cover various aspects, including medical, social, and functional well-being, to provide a comprehensive evaluation. The InterRAI-HC assessment includes 236 questions spanning 20 domains. Data collection is performed by assessors specifically trained and assessed by the Ministry of Health New Zealand, ensuring high data collection quality. Furthermore, yearly quality assessments ensure consistency (27). More detailed information concerning InterRAI can be found at https://interrai.org/.

### Participant Selection

**Figure 1.**
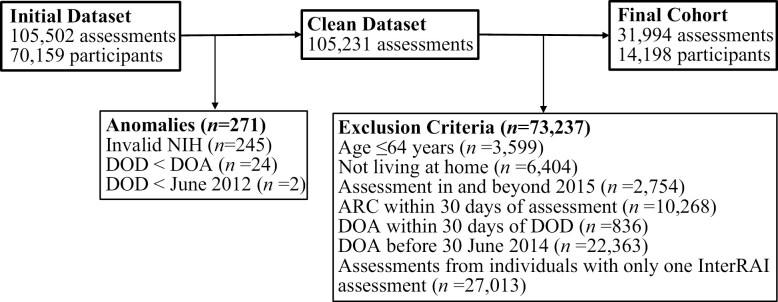
InterRAI participant selection flowchart*.* ARC = admission to residential care; DOA = date of admission; DOD = date of death.

For the current study, from InterRAI-HC, we isolated a retrospective condensed data set pertaining to a cohort of community-dwelling older adults aged ≥65 years with complex care needs, focusing on extracting key demographic and phenotypic data with minimal missing values. InterRAI participants included in this study exhibit high levels of morbidity, with over 20% of participants hospitalized 30 days prior to assessment. The assessment date for the patients was between 01/06/2012 and 30/06/2014. The timeframe and the fact that all participants are from the same country increases the homogenous nature of the sample in terms of clinical management. The full data set contained 105 502 assessments of 70 159 participants and 597 variables (28). The condensed data set, published in previous literature, contained 14 198 participants and 26 variables (29). The condensed dataset contained zero missing values. Figure 1 shows the participant selection flowchart. As all variables within the data set are categorical, the summary in Supplementary Table 1 describes them in terms of relative frequencies. The primary outcome was 30-day hospitalization, defined as any hospitalization related to any clinical event within 30 days. Hospital admission data were obtained from the complete InterRAI data set. The baseline assessment determined the 30-day hospital admission window. Questions A12, A14, N4, and S2 from the “interRAI Home Care Assessment Form” refer to hospitalization. The study contained several exclusions. First, 6 404 assessments were conducted on participants who did not live at home. Participants not living at home represent a different cohort, with higher levels of multimorbidity, different factors influencing hospitalization due to different functional and clinical support available in the nursing home, and a higher prevalence of advance care directives limiting hospital care. Second, in our analyses, we included participants who had undergone more than 1 interRAI assessment to analyze a homogenous population and mitigate potential selection bias.

### DBI Calculations

The DBI data were obtained from the New Zealand Pharms data set between 2012 and -2014, which is linked to interRAI (30). Pharms contains information regarding the drug name, dose, daily dose, base units, strength, frequency, therapeutic group, quantity prescribed and dispensed, and the provider type. Hospitalization data were obtained from the National Minimum Dataset, which contains information regarding the hospitalization start date, end date, and event type.

The DBI was calculated as follows:


$$\begin{array}{l} DBI=\sum \frac{D}{MDD+D} \end{array}$$

where *D* is the daily dose taken (estimated from dispensing data), and MDD is the minimum daily dose licensed for adults in New Zealand. The equation earlier considers all formulations of 1 medication. The patient’s DBI is the sum of the DBIs calculated for each medication they are dispensed.

### Data Preparation

The data set was unbalanced concerning 30-day hospitalization, with 2 857 having a hospitalization and 11 341 not having a hospitalization. Therefore, SMOTE (Synthetic Minority Oversampling Technique) was utilized to oversample the minority class. This was done to minimize the impact of an unbalanced data set which could obscure underlying patterns and associations, potentially leading to misleading conclusions. After SMOTE, the data set contained 22 864 observations. To assess predictive performance, the data set was split, 70:30, into training (*n* = 15 867) and validation cohorts (*n* = 6 997), respectively. For 11 523 observations, the 30-day-hospitalization flag was positive and 11 341 negative. All variables from the condensed data set were included in the analysis; these can be seen in Supplementary Table 1.

### ML Model Development and Validation

Variable selection was based on demographic variables and variables deemed clinically relevant for predicting short-term hospitalization in the available literature (31–38). Initially, all variables were converted to factors, and a reference factor level for each variable was set. The “hospitalization” variable was converted from a 4-level factor to a binary factor representing 30-day hospitalization as a Y/N. DBI was refactored into 1 of 2 subgroups (0–1 considered low and >1 high), in line with previous literature (20,30,39). Three classification models were developed and validated during the project: LR, RF, and XGB. The same seed (“7895”) was used for all models. A full binomial model was run using all variables using 100 times repeated cross-validation (2 repeats) for the LR model. For the RF model, Mtry was optimized to 6, and the remaining hyperparameters were left at default. For the XGB model, the independent variables in the training and validation data set were 1-hot encoded. The model was tuned using a tuning grid, optimizing for nrounds, max_depth, eta, gamma, colsample_bytree, min_child_weight, and subsample. All 3 models utilized all baseline variables. The LR, RF, and XGB models underwent 100 times repeated cross-validation (2 repeats) using the “Nimbus” high-performance computing unit at the University of Bath. Model performance was evaluated using area under the receiver-operating characteristics curve (AUC-ROC), accuracy, balanced accuracy, sensitivity, specificity, positive predictive value (PPV), negative predictive value (NPV), and F1 score. All results have been presented in interpretable formats, ensuring benefit to the clinician. All models were built and evaluated in R version 4.3.0. The ML code has been deposited in a GitHub repository: https://github.com/RobertOlender/ML_InterRAI_Hospitalisation_28092023

### Variable Importance

Variable importance plots were generated to visualize the importance of each variable in predicting the 30-day-hospitalization variable correctly. To achieve this, the change in accuracy in out-of-bag samples is obtained through permutation. For each variable, a reference level was set to establish the baseline, allowing better interpretation of the plots for the direction of effect.

## Results

Of the 3 ML models, as per Table 1, the RF model showed the greatest discriminatory power, predicting 30-day hospitalization with an accuracy of 92% (95% confidence interval [CI]: 0.9143, 0.9273). The XGB and LR models achieved a predictive accuracy of 82% (95% CI: 0.8144, 0.8327) and 67% (95%CI: 0.6570, 0.6795), respectively. The RF model significantly outperformed both XGB and LR models in terms of AUC-ROC (0.97, 0.90, 0.72, respectively), sensitivity (0.93, 0.78, and 0.69, respectively), and NPV (0.93, 0.81, and 0.66, respectively).

**Table 1.** Model Evaluation Statistics for the Cross-Validated RF, XGB, and LR Models Predicting 30-Day Hospitalization

Model	Accuracy (95% CI)	Balanced Accuracy	Sensitivity	Specificity	PPV	NPV	F1 Score
RF	0.9210 (0.9143, 0.9273)	0.9206	0.9344	0.9068	0.9134	0.9293	0.9238
XGB	0.8237 (0.8144, 0.8327)	0.8227	0.7814	0.8639	0.8451	0.8061	0.8120
LR	0.6683 (0.6570, 0.6795)	0.6677	0.6908	0.6446	0.6716	0.6646	0.6811

*Notes:* CI = confidence interval; NPV = negative predictive value; PPV = positive predictive value.

The confusion matrix (Table 2) provides insight into which cases the model classified as true positives, true negatives, false positives, and false negatives. The RF model achieved the highest overall accuracy, classifying 6 269 cases of 30-day hospitalization correctly. The RF model classified 229 cases as false negatives and 309 as false positives. In models predicting clinical events for preventative purposes, it is preferred for a model to have a higher false-positive than false-negative rate. This is because it is imperative not to miss high-risk patients at risk of 30-day hospitalization. Both the XGB and LR models had a higher PPV than NPV.

**Table 2.** Confusion Matrix for the Cross-Validated RF, XGB, and LR Models Predicting 30-Day Hospitalization

RF	Reference		XGB	Reference		LR	Reference	
Prediction	Y	N	Prediction	Y	N	Prediction	Y	N
Y	3 261	309	Y	2 592	475	Y	2 411	1 179
N	229	3 008	N	725	3 015	N	1 079	2 138

*Notes*: LR = Logistic Regression; RF = Random Forest; XGB =XGBoost.

**Figure 2.**
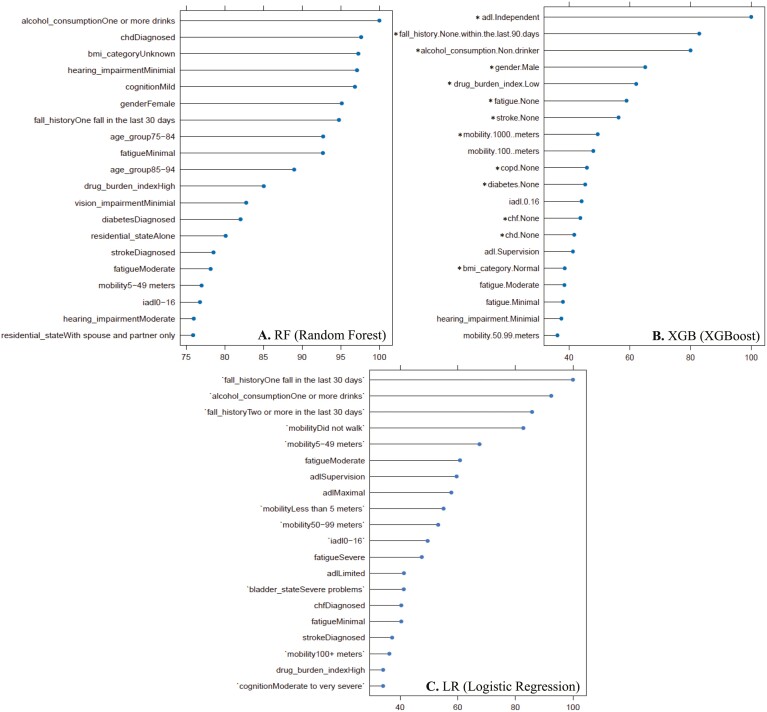
Variable importance plots for 3 models—(A) Random Forest, (B) XGBoost, (C) Logistic Regression—predicting 30-day hospitalization. Only the top 20 most important variables are displayed. Variables marked with an “*” represent the variable’s reference level. For a complete list of variables included in the models, please refer to Supplementary Table 1. Arbitrary scale for interpretability and comparability.

The top 20 most important variables for predicting the 30-day hospitalization are displayed in Figure 2. It should be noted that variable importance plots represent the aggregated importance scores by factor level, meaning the plot shows the top 20 most important variable levels out of a total of 84 variable factor levels. Additionally, all variables have a reference factor set, marked by an “*” in Figure 2, which suggests this variable factor level was important for predicting lack of 30-day hospitalization.

In all 3 models, the variable importance statistics show the DBI as important in predicting 30-day hospitalization. In the RF model, high DBI was the 10th most important variable. In the XGB model, low DBI was the fifth most important variable (predicting lack of 30-day hospitalization). In the LR model, high DBI was the 10th most important variable. Alcohol consumption was the most important variable in determining 30-day hospitalization. Other important variables include fall history, mobility, and ADLs (activities of daily living).

**Figure 3.**
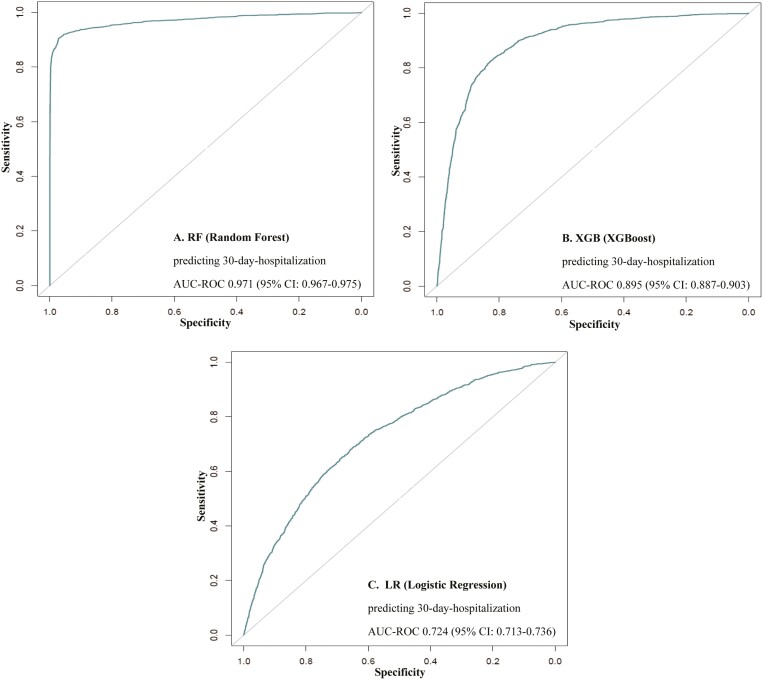
AUC-ROC of the 3 cross-validated ML models—(A) Random Forest; (B) XGBoost, (C) Logistic Regression—for predicting 30-day-hospitalization. 95% CI = 95% confidence interval; AUC-ROC = area under the receiver-operating characteristics curve.

The RF model achieved the highest AUC-ROC, followed by XGB and LR: 0.971, 0.895, and 0.724, respectively. All 3 ML models showed good discriminatory power. However, the RF model proved particularly powerful. AUC-ROC curves can be seen in Figure 3.

## Discussion

The potential applications of ML in geriatric medicine are vast and transformative. Accurately predicting unfavorable clinical outcomes such as 30-day hospitalization will allow clinicians to prioritize high-risk patients. By leveraging ML models that identify patients at high risk of 30-day hospitalization, clinicians can gain actionable insights into the modifiable risk factors affecting each patient. This, in turn, allows health systems to identify patients in need of urgent clinical review, and healthcare professionals to tailor their individual patient care strategies, aiming to enhance patients’ chances of a favorable outcome and effectively reduce the likelihood of hospitalization.

In this study, we developed 3 ML models to predict 30-day hospitalization in older adults with complex care needs. Our RF model emerged as the most effective, achieving a high AUC-ROC of 0.97 and accuracy of 92%. The XGB and LR models also performed commendably, with AUC-ROC scores of 0.90 and 0.72, respectively. These results compare favorably with several recent studies aimed at similar objectives. For example, Bories et al. developed and optimized 3 ML models to predict hospitalization due to bleeding in a sample of 7 462 participants prescribed oral anticoagulants. In their study, the RF, XGB, and Support Vector Machine models achieved accuracy levels of 0.64, 0.68, and 0.64, respectively (40). Verdu-Rotellar et al. utilized a multivariable LR model to predict 30-day hospitalization in a sample of 811 participants, achieving AUC-ROC scores of 0.73 and 0.89 in validation and derivation cohorts, respectively (41). Our RF and XGB models, in particular, appear to show superior discriminatory power when compared to these recently published models. For instance, Friz et al. predicted 30-day readmission in 3 079 participants using an adaptive boosting model, gradient-boosting model, XGB, and RF, with AUC-ROC scores of 0.803, 0.782, 0.776, and 0.786, respectively. Notably, all of these models outperformed the traditional LACE (Length of hospitalization, Acuity, Comorbidities, Emergency department visits) index, which achieved an AUC-ROC of 0.504 (42). Several other clinical outcomes have been predicted using RF, XGB, and LR models in recent literature, including but not limited to thrombolysis (43), 3-month giant cell arthritis flare-up (44), adverse events within 30 days of emergency department admission (45), and complications within 30 days of hospital admission (46). The models presented in the current study outperform most models from recent literature. High model performance can be attributed to several factors such as the high quality of data on which the models were trained, and the carefully selected predictors that have been linked to short-term hospitalizations in pervious literature. In summary, both our RF and XGB models demonstrate good discriminatory power and may offer significant advantages in predicting hospitalizations in older adults. Nevertheless, external validation in a cohort representative of the general population of older adults remains a crucial next step to confirm these promising findings.

Our ML models identified DBI and alcohol consumption as important variables for predicting 30-day hospitalization in older adults. Identifying these 2 variables is particularly interesting to clinicians, as they are potentially immediately-modifiable risk factors, while acknowledging that for some people, deprescribing and reducing alcohol consumption can take weeks or even years to complete. Regarding the DBI, deprescribing—reducing or eliminating medications with anticholinergic or sedative effects where the harm outweighs the benefit for the individual—is a clinically sound strategy, but it is often a challenging task that has been met with limited success (47). A study by Nishtala et al. showed that pharmacist-recommended dose changes for DBI-contributing drugs resulted in significantly decreased DBI (48). Regarding alcohol consumption, a link between alcohol consumption and hospitalization has been documented in the literature. Sacco et al. showed that rates of alcohol-related hospitalizations are increasing for older adults. The group recommended that training healthcare professionals to combat this issue is crucial (31). In a study by Kohli et al., alcohol-related and alcohol-withdrawal hospitalizations have led to increased length of stay, higher treatment costs, and greater functional decline (32). Additionally, rates of alcohol consumption among older adults (particularly females) have been shown to increase from 1997 to 2014 in the United States (33). This is relevant given that female gender ranked as the sixth most important variable in the RF model developed in the current study. InterRAI quantifies alcohol consumption as the highest number of drinks in any “single sitting” in the last 14 days. Patients identified as high risk should be specifically advised and educated on the negative effects of alcohol consumption on their health outcomes.

In contrast to the potentially immediately-modifiable DBI and alcohol consumption, our models also identified several risk factors that are modifiable with comprehensive geriatric assessment and rehabilitation but are less likely to be reduced in a timeframe that affects 30-day hospitalization. Fall history ranked as the seventh and second most important variable in the RF and XGB models and first in the LR model. Vaishya et al. linked falls with hospitalization and concluded that ensuring safe living environments is vital to preventing falls (35). Impaired mobility ranked as the 4th most important variable in the LR model and the 8th most important variable in the XGB model. Fisher et al. identified mobility as a physical biomarker for overall health and 30-day readmission (37). ADLs ranked as the first and fifth most important variable in the XGB and LR models, respectively. This is supported by a study by Nguyen et al., in which ADL impairment was associated with increased readmission in a cohort of patients with heart failure (34). Most of the less immediately-modifiable risk factors identified in our study have been linked with hospitalization in other literature, further supporting the variables identified by the models in this study. Gender is the sixth and fourth most important variable in the RF and XGB models, respectively. In a study by Gjestsen et al., gender, particularly male, was associated with a higher hospitalization rate (36).

### Impact of Our Study on the Clinical Care of Older Adults

Hospitalization, while sometimes unavoidable, carries inherent risks for older adults. A hospital stay can catalyze a cascade of additional complications for many older adults, including hospital-acquired pneumonia, delirium, falls, and functional decline. Thus, proactive measures to mitigate these risks—whether through judicious prescribing, lifestyle modifications, or closer outpatient monitoring—are crucial. In this context, ML models serve as a powerful ally for clinicians. They offer data-driven insights that can inform more personalized, precise, and preventive care strategies, helping to avert hospital admissions whenever possible and, consequently, the cascade of complications that can accompany them. In this study, we have developed and optimized 3 ML models for predicting 30-day hospitalization using the InterRAI data set, which is widely considered the gold standard due to its high data quality (49). Additionally, we identified DBI and alcohol consumption as important variables that are potentially immediately modifiable, in predicting 30-day hospitalization. Other risk factors, such as functional independence, mobility, and falls, may be modifiable over time, through comprehensive geriatric assessment and rehabilitation. As this field of study grows, large-scale studies deploying highly discriminatory ML models in external cohorts of older adults representative of the general population are needed. Identifying patients early will allow clinicians to proactively minimize the risk to the patient, ensuring a high standard of geriatric care and improving public health.

### Limitations and Strengths

This study has several limitations. First, the data set used to train and validate the models concerns people with complex care needs from New Zealand, bringing concerns about the generalizability to other older adult populations. Second, although our models underwent internal validation, they have not been validated in an external cohort of patients. However, we believe the RF model is appropriate for external validation, exhibiting a high accuracy, AUC-ROC, and NPV. Third, the 30-day-hospitalization outcome does not differentiate between emergency and elective admission. Fourth, the short-term mortality rate in the study population was not incorporated into the models, therefore, not accounting for the competing risk of death. Finally, The unbalanced and relatively small data set size can cause generalizability concerns. Although the data set was balanced for the primary outcome variable, it was not balanced for other variables. Large-scale external validation studies utilizing these models are necessary.

This study also had several strengths. First, interRAI is an exceptionally high-quality data set concerning older adults (49). All the geriatric assessments on which the data set is based are carried out by a healthcare professional trained to carry out the assessment. Second, the analyzed interRAI data set contained no missing values, so there was no need to employ data imputation techniques. Third, we performed 100 repeated cross-validations with 2 repeats for each ML model, minimizing the effects of model overfitting. Finally, we have fully documented our research, reporting multiple key model evaluation metrics. We have shared the complete R scripts in a GitHub repository to facilitate future external validation studies.

## Conclusion

As clinical databases continue to expand and evolve in complexity, so too does the potential of ML in enhancing geriatric care. A central application of these advanced tools lies in the identification of modifiable risk factors associated with 30-day hospitalization—a critical concern for clinicians dedicated to the well-being of older adults. As shown in our study, immediately-modifiable risk factors such as the DBI and alcohol consumption are of high clinical relevance, as clinicians can influence these variables and proactively lower the risk of 30-day hospitalization. Early recognition of these risk factors allows for timely and targeted interventions, aiming to actively minimize patients’ risk of hospitalization.

## Supplementary Material

glae130_suppl_Supplementary_Materials
