# Supplementary material for: Drug Burden Index Is a Modifiable Predictor of 30-Day Hospitalization in Community-Dwelling Older Adults With Complex Care Needs: Machine Learning Analysis of InterRAI Data
Source: J Gerontol A Biol Sci Med Sci. 2024 May 11;79(8):glae130. doi: 10.1093/gerona/glae130 (PMC11215698; doi:10.1093/gerona/glae130)
Supplement: glae130_suppl_Supplementary_Materials [file glae130_suppl_supplementary_materials.pdf]

Supplemental Material Table of Contents:

Table 1. Baseline participant characteristics. *Page 2-4*
Table 4. ML sensitivity analysis. *Page 5*

Table 1. Baseline participant characteristics of the condensed InterRAI dataset. Percentage rounded to 1 significant figure.

Variable	Full InterRAI Cohort (*n*)	%	30-Day-Hospitalization = Y (*n*)	%
**Total participants**	14,198		2,857	
**Cognition (impairment)**				
... None or minimal	8,148	57%	1,682	59%
... Mild	4,503	32%	855	30%
... Moderate	1,033	7%	231	8%
... Moderate to very severe	514	4%	89	3%
**Age Group (years)**				
... 65-74	2,124	15%	435	15%
... 75-84	6,003	42%	1,210	42%
... 85-94	5,627	40%	1,138	40%
... 95+	444	3%	74	3%
**Gender**				
... Female	8,866	62%	1,662	58%
... Male	5,332	38%	1,195	42%
**Ethnicity**				
... European	12,712	90%	2,510	88%
... Maori	629	4%	137	5%
... Other	857	6%	210	7%
**Marital State**				
... Married	12,637	89%	2,513	88%
... Other	1,561	11%	344	12%
**Residential State**				
... Alone	7,111	50%	1,358	48%
... With child only	1,499	11%	309	11%
... With non-relatives	125	1%	38	1%
... With relatives	303	2%	59	2%
... With spouse and partner only	4,625	33%	982	34%
... Other	535	4%	111	4%
**Bladder State**				
... Continent	8,893	63%	1,744	61%
... Good but not perfect	372	3%	137	5%
... Minor problems	1,320	9%	245	9%
... Some problems	1,197	8%	258	9%
... Severe problems	2,040	14%	378	13%
... Very Severe problems	361	3%	90	3%
…Out of control	15	<1%	5	<1%
**BMI Category**				
... Underweight	585	4%	142	5%
... Normal	3,540	25%	675	24%
... Overweight	2,690	19%	502	18%
... Obese	1,665	12%	310	11%
... Unknown	5,718	40%	1,228	43%
**ADL**				
... Independent	9,559	67%	1,523	53%
... Limited	1,348	9%	364	13%
... Dependent	311	2%	97	3%
... Extensive	885	6%	226	8%
... Maximal	237	2%	99	3%
... Supervision	1,858	13%	548	19%

Table 1. Baseline participant characteristics (cont.)

**IADL**				
... 0-16	4,188	29%	559	20%
... 17-28	4,439	31%	922	32%
... 29-38	3,407	24%	787	28%
... 39+	2,164	15%	589	21%
**Alcohol Consumption**				
... Non-drinker	10,867	77%	2,436	85%
... One or more drinks	3,331	23%	421	15%
**Smoking**				
... Non-smoker	13,534	95%	2,725	95%
... Smoker	664	5%	132	5%
**Hearing Impairment**				
... None	7,372	52%	1,495	52%
... Minimal	4,281	30%	881	31%
... Moderate	2,545	18%	481	17%
**Vision Impairment**				
... None	10,205	72%	2,048	72%
... Minimal	2,715	19%	548	19%
... Moderate	1,278	9%	261	9%
**Depression**				
... Absent	12,503	88%	2,513	88%
... Present	1,695	12%	344	12%
**Fall History**				
... None within the last 90 days	8,981	63%	1,466	51%
... Fell 31-90 days ago	1,647	12%	329	12%
... One fall in the last 30 days	2,199	15%	654	23%
... Two or more in the last 30 days	1,371	10%	408	14%
**Hospitalization**				
... N	11,341	80%	0	0%
... Y	2,857	20%	2,857	100%
**Stroke**				
... None	11,821	83%	2,260	79%
... Diagnosed	2,377	17%	597	21%
**COPD**				
... None	12,130	85%	2,334	82%
... Diagnosed	2,068	15%	523	18%
**Cancer**				
... None	12,631	89%	2,490	87%
... Diagnosed	1,567	11%	367	13%
**Fatigue**				
... None	4,638	33%	672	24%
... Minimal	5,066	36%	987	35%
... Moderate	3,325	23%	851	30%
... Severe	1,010	7%	296	10%
... Unable to commence any ADLs	159	1%	51	2%
**CHF**				
... None	11,884	84%	2,263	79%
... Diagnosed	2,314	16%	594	21%

Table 1. Baseline participant characteristics. (cont.)

**CHD**				
... None	9,716	68%	1,898	66%
... Diagnosed	4,482	32%	959	34%
**Diabetes**				
... None	11,305	80%	2,236	78%
... Diagnosed	2,893	20%	621	22%
**Mobility**				
... 1000+ meters	1,225	9%	85	3%
... 100+ meters	2,748	19%	375	13%
... 50-99 meters	2,403	17%	469	16%
... 5-49 meters	5,693	40%	1,296	45%
... Less than 5 meters	1,500	11%	373	13%
... Did not walk	629	4%	259	9%
**Drug Burden Index***				
... Low	5,262	37%	845	30%
... High	8,936	62%	2,012	70%

*Note. *The Drug_Burden_Index is defined as: (Low = 0-1, High = >1)*

Table 4. ML Sensitivity Analysis.

	AUC-ROC	Accuracy	Sensitivity	Specificity	PPV	NPV	F1 Score
**Full RF Model**	0.9710	0.9210	0.9344	0.9068	0.9134	0.9293	0.9238
**[RF - alcohol_consumption]***	0.9670	0.9144	0.9304	0.8975	0.9052	0.9245	0.9176
**[RF - alcohol_consumption] Δ performance**	-0.0040	-0.0066	-0.0040	-0.0093	-0.0082	-0.0048	-0.0062
**[RF - dbi]**	0.9683	0.9129	0.9289	0.8960	0.9038	0.9230	0.9162
**[RF - dbi] Δperformance**	-0.0027	-0.0081	-0.0055	-0.0108	-0.0096	-0.0063	-0.0076

	AUC-ROC	Accuracy	Sensitivity	Specificity	PPV	NPV	F1 Score
**Full XGB Model**	0.8950	0.8237	0.7814	0.8639	0.8451	0.8061	0.8120
**[XGB - ADL]****	0.8883	0.8209	0.7685	0.8708	0.8497	0.7983	0.8070
**[XGB - ADL] Δperformance**	-0.0067	-0.0028	-0.0129	0.0069	0.0046	-0.0078	-0.0050
**[XGB - dbi]**	0.8931	0.8262	0.7799	0.8702	0.8510	0.8062	0.8139
**[XGB - dbi] Δperformance**	-0.0019	0.0025	-0.0015	0.0063	0.0059	0.0001	0.0019

	AUC-ROC	Accuracy	Sensitivity	Specificity	PPV	NPV	F1 Score
**Full LR Model**	0.7240	0.6683	0.6908	0.6446	0.6716	0.6646	0.6811
**[LR - fall_history]*****	0.7076	0.6517	0.6779	0.6241	0.6549	0.6481	0.6662
**[LR - fall_history] Δperformance**	-0.0164	-0.0166	-0.0129	-0.0205	-0.0167	-0.0165	-0.0149
**[LR - DBI]**	0.7231	0.6678	0.6857	0.6491	0.6728	0.6625	0.6792
**[LR - DBI] Δperformance**	-0.0009	-0.0005	-0.0051	0.0045	0.0012	-0.0021	-0.0019

*Note. *alcohol_consumption was the most important variable in the RF model **ADL was the most important variable in the XGB model ***fall_history was the most important variable in the LR model.*
